# Клинические рекомендации "Синдром поликистозных яичников"

**DOI:** 10.14341/probl12874

**Published:** 2022-04-30

**Authors:** Л. В. Адамян, Е. Н. Андреева, Ю. С. Абсатарова, О. Р. Григорян, И. И. Дедов, Г. А. Мельниченко, Л. В. Сутурина, О. С. Филиппов, Е. В. Шереметьева, Г. Е. Чернуха, М. И. Ярмолинская

**Affiliations:** Национальный исследовательский центр акушерства, гинекологии и перинатологии им. В.И. Кулакова; Московский государственный медико-стоматологический университет им. А.И. Евдокимова; Национальный медицинский исследовательский центр эндокринологии; Московский государственный медико-стоматологический университет им. А.И. Евдокимова; Национальный медицинский исследовательский центр эндокринологии; Национальный медицинский исследовательский центр эндокринологии; Национальный медицинский исследовательский центр эндокринологии; Национальный медицинский исследовательский центр эндокринологии; Научный центр проблем здоровья семьи и репродукции человека; Первый Московский государственный медицинский университет им. И.М. Сеченова (Сеченовский Университет); Министерство здравоохранения Российской Федерации; Национальный медицинский исследовательский центр эндокринологии; Национальный исследовательский центр акушерства, гинекологии и перинатологии им. В.И. Кулакова; Научно-исследовательский институт акушерства, гинекологии и репродукции им. Д.О. Отта; Северо-Западный государственный медицинский университет им. И.И. Мечникова

**Keywords:** синдром поликистозных яичников, ановуляция, сахарный диабет 2 типа, комбинированные оральные контрацептивы, тестостерон, бесплодие

## Abstract

Синдром поликистозных яичников (СПЯ) — полигенное эндокринное расстройство, обусловленное как генетическими, так и эпигенетическими факторами. В зависимости от периода жизни женщины клиническая картина, диагностика, лечебная тактика заболевания различны. СПЯ имеет комплекс репродуктивных, метаболических и психологических особенностей. Целевая аудитория данных клинических рекомендаций — акушеры-гинекологи, эндокринологи, терапевты, врачи общей практики. В данных клинических рекомендациях все сведения ранжированы по уровням убедительности рекомендаций и достоверности доказательств в зависимости от количества и качества исследований по данной проблеме.

## Abstract (en)

Polycystic ovary syndrome (PCOS) is a polygenic endocrine disorder caused by both genetic and epigenetic factors. Depending on the period of a woman’s life, the clinical picture, diagnosis, and treatment tactics of the disease are different. PCOS has a complex of reproductive, metabolic and psychological characteristics. The target audience of these clinical recommendations are obstetrician-gynecologists, endocrinologists, general practitioners, general practitioners. In these clinical guidelines, all information is ranked according to the level of persuasiveness of recommendations and the reliability of evidence, depending on the number and quality of studies on this issue.

## СПИСОК СОКРАЩЕНИЙ

АД — артериальное давлениеАКТГ — адренокортикотропный гормонВРТ — вспомогательные репродуктивные технологииДЭАС — дегидроэпиандростерона сульфатИМТ — индекс массы телаИР — инсулинорезистентностьИСА — индекс свободных андрогеновКОК — комбинированные оральные контрацептивыЛГ — лютеинизирующий гормонМКБ-10 — международная классификация болезней 10 пересмотраМРТ — магнитно-резонансная томографиянВДКН — неклассическая форма врожденной дисфункции коры надпочечниковНТГ — нарушение толерантности к глюкозеОТ — окружность талииПГТТ — пероральный глюкозотолерантный тестПКЯ — поликистозные яичникиСД — сахарный диабетСОАС — синдром обструктивного апноэ снаСПЯ — синдром поликистозных яичниковССЗ — сердечно-сосудистые заболеванияГСПГ — глобулин, связывающий половые гормоныТТГ — тиреотропный гормонУДД — уровень достоверности доказательствУЗИ — ультразвуковое исследованиеУУР — уровень убедительности рекомендацийФСГ — фолликулостимулирующий гормон17ОНР — 17-оксипрогестеронХГЧ — хорионический гонадотропин человекаASRM — American Society for Reproductive MedicineESHRE — European Society of Human Reproduction and EmbryologyHOMA — Homeostasis model assessment

## 1. СИНДРОМ ПОЛИКИСТОЗНЫХ ЯИЧНИКОВ (СПЯ) — ПОЛИГЕННОЕ ЭНДОКРИННОЕ РАССТРОЙСТВО, ОБУСЛОВЛЕННОЕ КАК ГЕНЕТИЧЕСКИМИ, ТАК И ЭПИГЕНЕТИЧЕСКИМИ ФАКТОРАМИ. В ЗАВИСИМОСТИ ОТ ПЕРИОДА ЖИЗНИ ЖЕНЩИНЫ КЛИНИЧЕСКАЯ КАРТИНА, ДИАГНОСТИКА, ЛЕЧЕБНАЯ ТАКТИКА ЗАБОЛЕВАНИЯ РАЗЛИЧНЫ. СПЯ ИМЕЕТ КОМПЛЕКС РЕПРОДУКТИВНЫХ, МЕТАБОЛИЧЕСКИХ И ПСИХОЛОГИЧЕСКИХ ОСОБЕННОСТЕЙ [1].

СПЯ является одним из наиболее частых эндокринных расстройств у женщин репродуктивного возраста. СПЯ является фактором риска развития бесплодия, андрогензависимой дермопатии (акне, гирсутизма, алопеции), нарушений углеводного обмена (нарушения толерантности к глюкозе (НТГ), сахарного диабета (СД) 2 типа), дислипидемий, сердечно-сосудистой патологии, гиперпластических процессов эндометрия, нарушений психологического статуса (например, депрессии, тревожных расстройств, нарушений настроения), онкологических заболеваний (рака эндометрия, некоторых форм рака молочной железы), чему в значительной степени способствует наличие у 40–85% женщин с СПЯ избыточной массы тела или ожирения [2–4][98–100].

Несмотря на многочисленные исследования, до настоящего времени так и не удалось сформулировать единую концепцию патогенеза и этиологии СПЯ. В патогенезе заболевания условно можно выделить нарушения в 4 различных отделах нейроэндокринной системы, каждое из которых может претендовать на стартовую роль. Это нарушения на уровне гипоталамо-гипофизарной системы, яичников, надпочечников и периферических инсулинчувствительных тканей.

## 1.1. Эпидемиология заболевания или состояния (группы заболеваний или состояний)

В общей популяции женщин репродуктивного возраста распространенность синдрома составляет от 8 до 21%. Показатели распространенности СПЯ зависят от особенностей популяционной выборки [5].

## 1.2. Особенности кодирования заболевания или состояния (группы заболеваний или состояний) по Международной статической классификации болезней и проблем, связанных со здоровьем

Дисфункция яичников (E28):

E28.2 Синдром поликистоза яичников.

## 1.3 Классификация заболевания или состояния (группы заболеваний или состояний)

Европейским обществом репродукции (European Society of Human Reproduction and Embryology, ESHRE) и эмбриологии человека и Американским обществом репродуктивной медицины (American Society for Reproductive Medicine, ASRM) (Роттердам, 2003) [6] выделены основные критерии СПЯ: олигоановуляция, гиперандрогенемия (клиническая или биохимическая), поликистозная морфология яичников по данным ультразвукового исследования (УЗИ). Согласно ASRM/ESHRE (2003), International PCOS Network (2018), наличие любых 2 из 3 основных критериев определяет наличие определенного вида (фенотипа) СПЯ [1][5][7] (табл. 1).

**Table:** Таблица 1. Основные виды (фенотипы) СПЯTable 1. Main types (phenotypes) of PCOS

		Ановуляция	Гиперандрогения (клиническая биохимическая)	и/или	Поликистозная структура яичников по данным УЗИ
Вид (фенотип) («классический»)	А	+	+		+
Вид (фенотип) («ановуляторный)	В	+	+		
Вид (фенотип) («овуляторный»)	С		+		+
Вид (фенотип) («неандрогенный)	D	+			+

## 2. ДИАГНОСТИКА ЗАБОЛЕВАНИЯ ИЛИ СОСТОЯНИЯ (ГРУППЫ ЗАБОЛЕВАНИЙ ИЛИ СОСТОЯНИЙ), МЕДИЦИНСКИЕ ПОКАЗАНИЯ И ПРОТИВОПОКАЗАНИЯ К ПРИМЕНЕНИЮ МЕТОДОВ ДИАГНОСТИКИ

Диагностика СПЯ основана на результатах клинических и лабораторных проявлений гиперандрогении, оценке менструальной, овуляторной функции, а также морфологического строения яичников с помощью УЗИ.

Диагностические подходы отличаются у подростков и женщин репродуктивного возраста. У подростков СПЯ диагностируется при наличии клинической гиперандрогении и нерегулярного менструального цикла, при этом ультразвуковые критерии практически не используются. Также в этом разделе будут рассмотрены диагностические критерии сопутствующей патологии СПЯ, которая может возникать чаще, чем в общей популяции, а также являться следствием СПЯ.

Ультрасонографические критерии поликистозных яичников:

при использовании трансвагинальных датчиков с 8 MГц — наличие ≥20 фолликулов диаметром 2–9 мм в любом яичнике и/или увеличение объема любого яичника ≥10 см3 (при отсутствии желтого тела, кист или доминантных фолликулов);при использовании трансвагинальных датчиков с меньшими разрешающими характеристиками или при трансабдоминальном исследовании — увеличение объема любого яичника ≥10 см3 (при отсутствии желтого тела, кист или доминантных фолликулов).

## 2.1 Физикальное обследование

При наличии клинической гиперандрогении (акне, избыточный рост волос на теле и лице, выпадение волос на волосистой части головы) необходимо провести определенные оценочные методики.

Уровень убедительности рекомендаций (УУР) — С (уровень достоверности доказательств (УДД) — 5).

Комментарии: Распространенность гирсутизма при классическом фенотипе СПЯ достигает 75%.

Согласно последним рекомендациям, о гирсутизме, как правило, свидетельствует сумма баллов по модифицированной Шкале Ферримана–Голлвея ≥4–6, однако имеются расовые особенности оценки гирсутизма [1].

У некоторых представительниц европеоидной и негроидной рас патогномоничным является повышение значения суммы баллов по указанной шкале ≥8. У представительниц Юго-Восточной Азии диагностически значимо повышение суммы баллов по данной шкале ≥3 [12]. Более выраженный гирсутизм характерен для женщин Ближнего Востока, Латинской Америки и Средиземноморья [1]. Однако степень гирсутизма при СПЯ не всегда коррелирует со степенью избытка андрогенов. Тяжелый гирсутизм может наблюдаться при незначительном повышении уровня андрогенов в сыворотке крови, а значительное повышение показателей не всегда сопровождается гирсутизмом. Это несоответствие между уровнем гормонов и степенью выраженности гирсутизма отражает разную индивидуальную чувствительность ткани-мишени к этим гормонам.

УУР — С (УДД — 5).

Комментарии: Нет валидированных оценочных шкал для определения степени тяжести угревых высыпаний. Шкала Людвига предпочтительна для оценки степени выраженности алопеции.

Наличие акне и алопеции не является надежным критерием гиперандрогении.

У подростков в качестве клинического признака гиперандрогении рассматривается только выраженное акне.

УУР — С (УДД — 4).

Комментарии: К клиническим маркерам инсулинорезистентности (ИР) у пациенток с СПЯ относится черный акантоз (папиллярно-пигментная дистрофия кожи в виде локализованных участков бурой гиперпигментации в области кожных складок, чаще шеи, подмышечных впадин, паховой области, которые гистологически характеризуются гиперкератозом и папилломатозом).

Комментарии: ИМТ вычисляется по формуле:

ИМТ (кг/м2) = масса тела (кг)/рост2 (м2).

Повышение ИМТ при СПЯ встречается чаще, чем в общей популяции, что в 4 раза увеличивает риск СД 2 типа в этой популяции [90].

Ожирение при СПЯ:

УУР — С (УДД– 5).

Комментарии: Показателем абдоминального (висцерального или мужского) типа ожирения (с которым и связаны более высокие риски нарушений углеводного обмена и сердечно-сосудистых заболеваний) у женщин является ОТ>80 см. В Японии используют значения 85 см для мужчин и 90 см для женщин [101]. Женщины с СПЯ чаще имеют абдоминальное ожирение, которое ассоциировано с метаболическими заболеваниями. Абдоминальное ожирение напрямую связано с ИР. Измерение ОТ — информативный и простой антропометрический метод, позволяющий выявить метаболические нарушения, поскольку этот показатель прямо коррелирует с количеством абдоминального жира.

## 2.2 Лабораторные диагностические исследования

УУР — В (УДД — 2).

Комментарии: ИСА — это показатель соотношения общего тестостерона к глобулину, связывающему половые гормоны (ГСПГ). Для расчета ИСА используют формулу:

ИСА = общий тестостерон (нмоль/л) / ГСПГ(нмоль/л) х 100.

Нормальное значение ИСА у женщин репродуктивного периода — 0,8–11%.

Для расчета биодоступного тестостерона необходимо, наряду с тестостероном, определение уровня альбумина сыворотки крови.

УУР — В (УДД — 3).

Комментарии: При интерпретации показателей тестостерона необходимо руководствоваться референсными интервалами, используемыми лабораторией.

УУР — C (УДД– 5).

УУР — C (УДД– 5).

Комментарии: Данные показатели являются вспомогательными маркерами биохимической гиперандрогении при СПЯ и не должны использоваться на первом этапе диагностики.

УУР — C (УДД — 5).

Комментарии: Если необходимо проведение диагностических проб, то нужно отменить препараты на 3 мес. На время отмены КОК (по АТХ — Прогестагены и эстрогены (фиксированные сочетания)) женщинам, не планирующим беременность, необходимо рекомендовать негормональные методы контрацепции.

УУР — В (УДД — 2).

Рекомендуется проводить 2-часовой ПГТТ с 75 г глюкозы пациенткам с СПЯ при наличии факторов риска (ИМТ>25 кг/м2 (или >23 кг/м2 у азиаток), гипергликемия натощак, нарушение толерантности к углеводам или гестационный диабет в анамнезе, отягощенный наследственный анамнез по СД 2 типа, принадлежность к этнической группе высокого риска) [1][2][4][7][21–23].

УУР — C (УДД — 5).

Комментарии: У женщин репродуктивного возраста с СПЯ чаще, чем в общей популяции, развиваются нарушения углеводного обмена (в 5 раз — в Азии, в 4 раза — в США и в 3 раза — в Европе), независимо от ожирения, но усугубляются его наличием.

Оценку показаний к проведению ПГТТ у пациенток с СПЯ необходимо проводить на первичной консультации, на этапе прегравидарной подготовки и в период беременности между 24 и 28-й неделями (при отсутствии прегестационного сахарного диабета).

УУР — С (УДД — 5).

УУР — В (УДД — 2).

УУР — C (УДД — 5).

Комментарии: Золотым стандартом диагностики ИР является эугликемический гиперинсулинемический клэмп-тест с внутривенным введением инсулина и одновременной инфузией глюкозы для поддержания стабильного уровня гликемии. Упрощенной моделью клэмп-теста является внутривенный глюкозотолерантный тест, основанный на многократном определении гликемии и инсулина крови. Однако эти методы являются инвазивными, трудоемкими и дорогостоящими, что не позволяет широко использовать их в клинических исследованиях. Значения индексов HOMA и Caro существенно зависят от применяемого метода определения концентрации инсулина. Индекс HOMA-IR (в норме менее 3,9) определяется по формуле:

Уровень глюкозы натощак (ммоль/л) х Уровень инсулина натощак (мЕд/л)/22,5.

Индекс отношение глюкозы (в ммоль/л) к инсулину (в мкМЕ/мл) в плазме крови натощак. Нормальное значение ≥,33.

## 2.3. Инструментальные диагностические исследования

УУР — А (УДД — 2).

Комментарии: Рекомендовано использовать ультрасонографические критерии поликистозных яичников:

при использовании трансвагинальных датчиков с 8 MГц — наличие ≥20 фолликулов диаметром 2–9 мм в любом яичнике и/или увеличение объема любого яичника ≥10 см3 (при отсутствии желтого тела, кист или доминантных фолликулов);при использовании трансвагинальных датчиков с меньшими разрешающими характеристиками или при трансабдоминальном исследовании — увеличение объема любого яичника ≥10 см3 (при отсутствии желтого тела, кист или доминантных фолликулов).

При наличии желтого тела, кист или доминантных фолликулов УЗИ выполняется повторно, после спонтанной или индуцированной менструации.

УУР — С (УДД — 5).

УУР — С (УДД — 5).

Комментарии: Данные критерии не следует применять у женщин, получающих КОК.

## 2.4 Иные диагностические исследования

УУР — C (УДД — 5).

УУР — C (УДД — 5).

Комментарии: При регулярных менструальных циклах возможна оценка овуляторной функции яичников по данным УЗИ органов малого таза на 21–22-й день менструального цикла. Уровень прогестерона <3 нг/мл в середине лютеиновой фазы свидетельствует об отсутствии овуляции, <10 нг/мл или сумма измерений в 3 последовательных циклах <30 нг/мл может свидетельствовать о неполноценной лютеиновой фазе менструального цикла.

УУР — B (УДД — 2).

Комментарии: Пациентки с диагностированным СОАС направляются в специализированное лечебное учреждение.

ОЦЕНКА РИСКА СЕРДЕЧНО-СОСУДИСТЫХ ЗАБОЛЕВАНИЙ (ССЗ) У ЖЕНЩИН С СПЯ

УУР — С (УДД– 3).

УУР — С (УДД — 3).

УУР — С (УДД — 3).

Комментарии: К группе риска относят женщин с СПЯ при наличии хотя бы одного из следующих факторов: ожирение (особенно абдоминальное), курение, гипертензия, гиподинамия, дислипидемия, субклинический атеросклероз, нарушение толерантности к глюкозе, семейный анамнез по ранним ССЗ (менее 55 лет у родственников мужского пола, менее 65 лет — у родственниц женского пола).

УУР — В (УДД 2)

## 3. ЛЕЧЕНИЕ, ВКЛЮЧАЯ МЕДИКАМЕНТОЗНУЮ И НЕМЕДИКАМЕНТОЗНУЮ ТЕРАПИИ, ДИЕТОТЕРАПИЮ, ОБЕЗБОЛИВАНИЕ, МЕДИЦИНСКИЕ ПОКАЗАНИЯ И ПРОТИВОПОКАЗАНИЯ К ПРИМЕНЕНИЮ МЕТОДОВ ЛЕЧЕНИЯ

Цели лечения: устранение проявлений андрогензависимой дерматопатии, нормализация массы тела и коррекция метаболических нарушений, регуляция менструального цикла для профилактики гиперплазии эндометрия, восстановление овуляторного менструального цикла и фертильности, предупреждение поздних осложнений СПЯ. Индивидуальный план ведения пациентки составляется с учетом основных жалоб, репродуктивных установок, наличия риска сердечно-сосудистых заболеваний и прочих факторов.

КОК, метформин и другие фармакологические препараты при СПЯ используются off label (без официальных показаний в инструкции), однако имеется множество исследований, подтверждающих их эффективность. Врачи должны информировать пациенток и обсуждать эффективность, возможные побочные эффекты и последствия терапии для выработки персонализированной тактики ведения.

## 3.1. Консервативное лечение

УУР — А (УДД — 1).

Комментарии: Достижимые цели, такие как потеря веса на 5–10% в течение 6 мес у пациенток с избыточным весом, приводят к значительным клиническим улучшениям. Снижение массы тела на фоне модификации образа жизни у пациенток с СПЯ способствует нормализации менструальной функции и улучшению ряда метаболических показателей (преимущественно — углеводного обмена), однако ответ имеет индивидуальный характер. При СПЯ недостаточно доказательств предпочтения какой-либо конкретной диеты. Важно адаптировать диетические изменения в пищевых привычках пациентки с применением гибкого и индивидуального подхода по снижению калорийности питания и избегать излишне ограничительных и несбалансированных диет. Физическая активность у взрослых 18–64 лет должна составлять минимум 150 минут в неделю физической активности средней интенсивности, или 75 минут в неделю высокой интенсивности, или эквивалентная комбинация обоих, включая упражнения на укрепление мышц в течение 2 дней в неделю, не следующих подряд.

УУР — В (УДД — 1).

Комментарии: Женщинам с СПЯ, не заинтересованным в беременности, рекомендуются любые методы контрацепции с учетом критериев приемлемости контрацепции ВОЗ [65]. При применении кгК у большинства пациенток с СПЯ польза превышает риски. Они не оказывают негативного влияния на фертильность пациентки в будущем. Эффективность кгК обусловлена подавлением секреции ЛГ, что приводит к снижению продукции овариальных андрогенов; эстрогенный компонент кгК способствует повышению уровней ГСПГ, что, в свою очередь, снижает уровень свободно циркулирующего тестостерона; прогестин в составе кгК может осуществлять конкурентное взаимодействие с 5α-редуктазой на уровне рецепторов к андрогенам. Кроме того, кгК снижают продукцию надпочечниковых андрогенов, по-видимому, за счет подавления продукции АКТГ. Для терапии может использоваться любой кгК с любой дозой эстрогенов, однако препараты, содержащие 35 мкг этинилэстрадиола и ципротерон, не должны рассматриваться как препараты первой линии при СПЯ из-за побочных эффектов, включая риск венозных тромбоэмболий. Следует выбирать препарат с минимально-эффективной дозой этинилэстрадиола (20–30 мкг), гестаген же может быть любой, однако стоит принимать во внимание метаболическую нейтральность гестагенов при подборе лечения. Необходимо также учитывать наличие таких ассоциированных с СПЯ состояний, как избыточный вес и ожирение, гиперлипидемия и артериальная гипертензия. Результаты исследований показывают, что у пациенток с СПЯ в 3 раза чаще встречается гипергомоцистеинемия, являющаяся фактором риска сердечно-сосудистой патологии. В большинстве случаев повышение уровня гомоцистеина — это результат дефицита фолатов в организме, поэтому пациенткам с СПЯ могут быть рекомендованы КОК с фолатами [92–96][103–107].

УУР — B (УДД — 1).

УУР — В (УДД — 1).

Комментарии: При назначении антиандрогенов необходима надежная контрацепция. ципротерон (50–100 мг в сутки) в циклическом или непрерывном режиме можно использовать у женщин в качестве лечения выраженных явлений андрогенизации (гирсутизма, акне) [1][122][123]. Спиронолактон (от 50 до 200 мг в сутки) может быть рекомендован для лечения акне [2][122]. Финастерид (2,5–5 мг в сутки) и флутамид (250–500 мг в сутки) не зарегистрированы в России для лечения гирсутизма у женщин, хотя они могут быть эффективны [111]. Флутамид может обладать гепатотоксичностью, что следует принимать во внимание при подборе терапии.

УУР — В (УДД — 1).

Комментарии: Метформин назначается в дозе 500 мг в сутки с постепенным еженедельным повышением по 500 мг, максимальная суточная доза — 1500 мг [59][66][68][69].

Рекомендуется применять метформин в дополнение к модификации образа жизни у женщин с СПЯ и ИМТ≥25 кг/м2 для контроля веса и улучшения метаболических исходов, а также подросткам «группы риска» или с установленным диагнозом СПЯ [66][69].

УУР — A (УДД — 2).

Комментарии: Метформин может оказать терапевтический эффект в группах женщин с высоким метаболическим риском (факторы риска сахарного диабета, наличие нарушенной толерантности к глюкозе или определенные этнические группы высокого риска). При назначении метформина необходимо учитывать побочные эффекты со стороны желудочно-кишечного тракта, которые, как правило, зависят от дозы. Поэтому необходимо начинать с низкой дозы, с шагом 500 мг 1–2 раза в неделю. Препараты с пролонгированным высвобождением могут минимизировать побочные эффекты. Следует помнить, что длительное применение метформина может приводить к снижению витамина B12, поэтому назначение данной терапии должно обсуждаться с пациенткой. Следует информировать женщину о возможной эффективности, рисках и побочных эффектах этого лечения.

УУР — В (УДД — 1).

Рекомендуется использовать фармакотерапию ожирения у пациенток с СПЯ и ИМТ≥30 кг/м2 или ИМТ≥27 кг/м2 при наличии хотя бы одного из следующих осложнений: артериальная гипертензия, дислипидемия, СД 2 типа, СОАС [1][66][70][71].

УУР — A (УДД — 2).

Комментарии: Поведенческая терапия с целью уменьшения потребления пищи и увеличения физической активности является обязательным условием проводимого лечения. Оценку эффективности лекарственной терапии ожирения следует проводить спустя 3 мес от начала лечения. Неэффективным может считаться снижение массы тела менее чем 5% исходной в течение 3 мес.

При выборе препарата для лечения ожирения при СПЯ нужно принимать во внимание следующие данные.

У женщин с СПЯ, ИР, избыточным весом или ожирением при одновременном применении орлистата или метформина с КОК комбинация КОК+орлистат ассоциирована с более эффективным снижением веса и уровней липидов при лучшей переносимости лечения.

При применении лираглутида регистрируется большая потеря веса, чем при использовании орлистата и метформина. Изолированное применение лираглутида, его сочетания с метформином и изолированное использование метформина приводит, в отличие от орлистата, к уменьшению ОТ.

Исследования сибутрамина при СПЯ свидетельствуют о его эффективности, однако перед его применением требуется тщательная оценка кардиоваскулярного риска.

См. соответствующие клинические рекомендации «Ожирение».

Рекомендуется применение бариатрической хирургии при СПЯ и ИМТ ≥40 кг/м2 или ≥35 кг/м2 при наличии осложнений, связанных с ожирением [66].

УУР — A (УДД — 2).

УУР — B (УДД — 1).

Комментарии: Цель лечения пациенток с СПЯ — восстановление овуляторных менструальных циклов.

Модификация образа жизни, в частности, лечение ожирения, должны предшествовать индукции овуляции при СПЯ. Перед индукцией овуляции у женщин с СПЯ должны быть исключены другие причины бесплодия в паре (трубно-перитонеальный, мужской факторы).

Согласно международным клиническим рекомендациям, препаратом первой линии для лечения ановуляторного бесплодия рекомендован нестероидный ингибитор ароматазы — летрозол [1], однако в России этот препарат может быть рекомендован только с подписанием информированного добровольного согласия. Стимуляция овуляции летрозолом проводится в дозе 2,5 мг в сутки с 3-го по 7-й или с 5-го по 9-й дни менструального цикла, в случае отсутствия овуляции в следующем цикле стимуляции возможно увеличение дозы #летрозола до 5 мг в сутки. Максимальная дозировка #летрозола в протоколе стимуляции овуляции составляет 7,5 мг в сутки [124]. Согласно данным проведенных РКИ и метаанализов, #летрозол в 1,5 раза эффективнее кломифена в достижении овуляции, наступлении беременности и живорождения без увеличения рисков многоплодной беременности или невынашивания [125].

При отборе пациенток для применения кломифена рекомендуется принимать во внимание ИМТ, возраст пациентки, наличие прочих факторов бесплодия. Кломифен назначается по 50–100 мг в день, в течение 5 дней, начиная со 2–5-го дня спонтанного или индуцированного менструального цикла. Стартовая доза составляет, как правило, 50 мг в день, максимальная суточная доза — 150 мг. Эффективность стимуляции овуляции достигает 70–80%, частота зачатия — 22% на цикл. Лечение кломифеном проводится, как правило, в течение не более 6 овуляторных циклов. Кумулятивная частота рождения живых детей в расчете на 6 циклов индукции овуляции составляет 50–60% [91]. Повышенный индекс свободного тестостерона и ИМТ, наличие аменореи, увеличенный объем яичников являются предикторами неэффективного применения кломифена [92].

УУР — В (УДД — 1).

Комментарии: Для преодоления резистентности к КЦ его можно комбинировать с метформином для повышения шансов на беременность (повышение частоты овуляции и зачатия в 1,6 раза, частоты живорождений — в 1,2 раза). Если #метформин используется для индукции овуляции у женщин с СПЯ, ожирением (ИМТ ≥30 кг/м2), ановуляторным бесплодием и отсутствием других факторов бесплодия, можно добавить КЦ для улучшения овуляторной функции и вероятности беременности.

УУР — B (УДД — 1).

Комментарии: Гонадотропины могут использоваться в сочетании с #метформином у женщин с СПЯ, ановуляторным бесплодием, резистентностью к КЦ и отсутствием других факторов бесплодия для улучшения овуляторной функции, увеличения вероятности наступления беременности и рождаемости. Продолжительность использования гонадотропинов не должна превышать 6 циклов. При проведении стимуляции гонадотропинами рекомендуется мониторировать овариальный ответ.

## 3.2 Хирургическое лечение

УУР — В (УДД — 1).

Комментарии: Эффективность лапароскопического дриллинга и применения гонадотропинов сопоставимы. Монополярная электрокаутеризация и лазер применяются с одинаковой эффективностью [67][108]. Для достижения эффекта при СПЯ достаточно 4 пункций яичника, с большим их числом ассоциировано возрастание преждевременной овариальной недостаточности. У 50% пациентов после лапароскопии требуется индукция овуляции. Если через 12 нед после лапароскопии овуляция отсутствует, следует использовать стимуляцию КЦ, а через 6 мес применения КЦ возможно применение гонадотропинов.

УУР — В (УДД — 1).

УУР — С (УДД — 4).

Комментарии: Соотношение риска и пользы данной операции в настоящее время не определено. Требуются дальнейшие исследования. Рекомендуется использование надежной контрацепции в течение 12 мес после операции.

## 4. МЕДИЦИНСКАЯ РЕАБИЛИТАЦИЯ, МЕДИЦИНСКИЕ ПОКАЗАНИЯ И ПРОТИВОПОКАЗАНИЯ К ПРИМЕНЕНИЮ МЕТОДОВ РЕАБИЛИТАЦИИ

В период послеоперационной реабилитации необходимы ограничение тяжелых физических нагрузок, профилактика запоров. При эффективном хирургическом лечении СПЯ наступление беременности возможно уже в течение 3 мес после лапароскопической операции.

## 5. ПРОФИЛАКТИКА И ДИСПАНСЕРНОЕ НАБЛЮДЕНИЕ, МЕДИЦИНСКИЕ ПОКАЗАНИЯ И ПРОТИВОПОКАЗАНИЯ К ПРИМЕНЕНИЮ МЕТОДОВ ПРОФИЛАКТИКИ

УУР — С (УДД — 4).

## 6. ОРГАНИЗАЦИЯ ОКАЗАНИЯ МЕДИЦИНСКОЙ ПОМОЩИ

Показания для госпитализации в медицинскую организацию.

Показания к выписке пациентки из медицинской организации.

## 7. ДОПОЛНИТЕЛЬНАЯ ИНФОРМАЦИЯ (В ТОМ ЧИСЛЕ ФАКТОРЫ, ВЛИЯЮЩИЕ НА ИСХОД ЗАБОЛЕВАНИЯ ИЛИ СОСТОЯНИЯ)

## 7.1 Дифференциальная диагностика

Наиболее часто встречающиеся нозологии, протекающие под маской СПЯ, представлены в приложении Г3.

УУР — C (УДД — 5).

Комментарии: Критериями диагностики манифестного гипотиреоза являются повышение уровня тиреотропного гормона (ТТГ) выше его нормальных значений и снижение концентраций свободной фракции тироксина. Снижение уровня ТТГ менее нижней границы нормы (обычно менее 0,1 мЕД/л) свидетельствует о гипертиреозе. Рекомендуется как минимум двукратное проведение лабораторного исследования уровня пролактина.

УУР — B (УДД — 2).

Комментарии: Для диагностики неклассической формы забор крови на 17ОНР проводят рано утром в фолликулярную фазу цикла (не позднее 5–7 дня), при аменорее — в любой день, строго вне беременности. Нормой считаются показатели менее 6 нмоль/л или менее 2 нг/мл, ниже этих уровней нВДКН практически не встречается. Следует помнить, что референсные значения, которые приводятся различными лабораториями, обычно отличаются и могут быть значительно ниже указанных «отрезных точек» для диагностики нВДКН. В случае значений базального 17ОНР более 30 нмоль/л или 10 нг/мл диагноз ВДКН считается подтвержденным, и дополнительной диагностики не требуется. При пограничных значениях 17ОНР (6–30 нмоль/л или 2–10 нг/мл — так называемая «серая зона»), выявленных минимум при двукратном определении, необходимо КР82 13 проводить дополнительный стимулирующий тест с тетракозактидом — синтетическим аналогом АКТГ, что является золотым стандартом диагностики ВДКН во всем мире. При сомнительных результатах определения 17ОНР, невозможности проведения пробы с тетракозактидом, а также в целях генетического консультирования далее рекомендуется проводить генотипирование.

## 7.2 ВРТ и СПЯ

УУР — В (УДД — 2).

Комментарии: У пациенток с СПЯ при применении ВРТ высок риск гиперстимуляции яичников. Частота наступления клинической беременности на лечебный цикл у женщин с СПЯ составляет 35%, что сопоставимо с таковой у пациенток без СПЯ. Рекомендуется проводить перенос 1 эмбриона.

Предпочтителен протокол с антагонистами ГнРГ (по АТХ — Антигонадотропины) для уменьшения длительности стимуляции, дозы гонадотропинов и частоты синдрома гиперстимуляции яичников (СГЯ). Применение агонистов ГнРГ (по АТХ — Аналоги гонадотропин-рилизинг-гормона) в качестве триггера финального созревания ооцитов следует рекомендовать при повышенном риске СГЯ или в случае отсроченного переноса эмбрионов.

УУР — В (УДД — 1).

Комментарии: Метформин может снизить риски гиперстимуляции, однако значимо не влияет на уровень живорождения. Метформин назначается в дозе от 1000 до 2500 мг в сутки [1][83–86]. При применении in vitro maturation (IVM) не характерно развитие синдрома гиперстимуляции яичников, что позволяет рассматривать данный метод как альтернативный.

## 7.3 Акушерские аспекты СПЯ

УУР — В (УДД — 2).

Комментарии: Женщины с СПЯ представляют собой группу риска по развитию неблагоприятных исходов беременности. Частота ГСД, артериальной гипертензии, преэклампсии, согласно результатам мета-анализов, повышается в 3–4 раза. Риск осложненного течения беременности выше у женщин с «классическим» фенотипом СПЯ. Предконцепционная подготовка должна включать: отказ от курения, модификацию образа жизни, использование фолиевой кислоты. При естественном наступлении беременности у женщин с СПЯ частота выкидышей не увеличена, вне зависимости от наличия или отсутствия ожирения. Уровень невынашивания после индукции овуляции сопоставим с таковым при прочих формах бесплодия.

УУР — В (УДД — 2)

## ДОПОЛНИТЕЛЬНАЯ ИНФОРМАЦИЯ

Источники финансирования. Работа выполнена по инициативе авторов без привлечения финансирования.

Конфликт интересов. Авторы декларируют отсутствие явных и потенциальных конфликтов интересов, связанных с содержанием настоящей статьи.

Участие авторов. Все авторы одобрили финальную версию статьи перед публикацией, выразили согласие нести ответственность за все аспекты работы, подразумевающую надлежащее изучение и решение вопросов, связанных с точностью или добросовестностью любой части работы.

## ПРИЛОЖЕНИЕ А2.

## МЕТОДОЛОГИЯ РАЗРАБОТКИ КЛИНИЧЕСКИХ РЕКОМЕНДАЦИЙ

Целевая аудитория данных клинических рекомендаций:

В данных клинических рекомендациях все сведения ранжированы по уровню убедительности рекомендаций и достоверности доказательств в зависимости от количества и качества исследований по данной проблеме (табл. 3–5).

**Table:** Таблица 3. Шкала оценки уровней достоверности доказательств (УДД) для методов диагностики (диагностических вмешательств)Table 3. Evidence Confidence Level (ER) rating scale for diagnostic methods (diagnostic interventions)

УДД	Расшифровка
1	Систематические обзоры исследований с контролем референсным методом или систематический обзор рандомизированных клинических исследований с применением мета-анализа
2	Отдельные исследования с контролем референсным методом или отдельные рандомизированные клинические исследования и систематические обзоры исследований любого дизайна, за исключением рандомизированных клинических исследований, с применением мета-анализа
3	Исследования без последовательного контроля референсным методом или исследования с референсным методом, не являющимся независимым от исследуемого метода или нерандомизированные сравнительные исследования, в том числе когортные исследования
4	Несравнительные исследования, описание клинического случая
5	Имеется лишь обоснование механизма действия или мнение экспертов

**Table:** Таблица 4. Шкала оценки уровней достоверности доказательств (УДД) для методов профилактики, лечения и реабилитации (профилактических, лечебных, реабилитационных вмешательств)Table 4. Evidence Reliability Rating Scale (ERR) for prevention, treatment and rehabilitation methods (preventive, curative, rehabilitative interventions)

УДД	Расшифровка
1	Систематический обзор РКИ с применением мета-анализа
2	Отдельные РКИ и систематические обзоры исследований любого дизайна, за исключением РКИ, с применением мета-анализа
3	Нерандомизированные сравнительные исследования, в т.ч. когортные исследования
4	Несравнительные исследования, описание клинического случая или серии случаев, исследования «случай-контроль»
5	Имеется лишь обоснование механизма действия вмешательства (доклинические исследования) илимнение экспертов

**Table:** Таблица 5. Шкала оценки уровней убедительности рекомендаций (УУР) для методов профилактики, диагностики, лечения и реабилитации (профилактических, диагностических, лечебных, реабилитационных вмешательств)Table 5. Scale for assessing the levels of persuasiveness of recommendations (RCR) for methods of prevention, diagnosis, treatment and rehabilitation (prophylactic, diagnostic, therapeutic, rehabilitation interventions)

УДД	Расшифровка
A	Сильная рекомендация (все рассматриваемые критерии эффективности (исходы) являются важными, все исследования имеют высокое или удовлетворительное методологическое качество, их выводы по интересующим исходам являются согласованными)
B	Условная рекомендация (не все рассматриваемые критерии эффективности (исходы) являются важными, не все исследования имеют высокое или удовлетворительное методологическое качество и/или их выводы по интересующим исходам не являются согласованными)
C	Слабая рекомендация (отсутствие доказательств надлежащего качества (все рассматриваемые критерии эффективности (исходы) являются неважными, все исследования имеют низкое методологическое качество и их выводы по интересующим исходам не являются согласованными)

Порядок обновления клинических рекомендаций.

Механизм обновления клинических рекомендаций предусматривает их систематическую актуализацию — не реже чем один раз в три года, а также при появлении новых данных с позиции доказательной медицины по вопросам диагностики, лечения, профилактики и реабилитации конкретных заболеваний, наличии обоснованных дополнений/замечаний к ранее утверждённым КР, но не чаще 1 раза в 6 месяцев.

## ПРИЛОЖЕНИЕ Г1.

## ШКАЛА ФЕРРИМАНА-ГАЛЛВЕЯ

Название на русском языке: Шкала Ферримана-Галлвея Оригинальное название (если есть): Ferriman gallwey scoreИсточник (официальный сайт разработчиков, публикация с валидацией): Yildizetal., 2010Тип (подчеркнуть): шкала оценкиНазначение: Оценка степени выраженности оволосенияСодержание (шаблон): Оценивается степень выраженности оволосения в 9 областях тела по 4-балльной шкалеКлюч (интерпретация): Сумма балоов по всем областям называется гирсутным числом.Пояснения: Рекомендуется не прибегать к депиляции или удалять волосы с помощью воска в течение, как минимум, 4-х недель и избегать сбривания волос в течение не менее 5 дней до проведения исследования.

**Figure:**
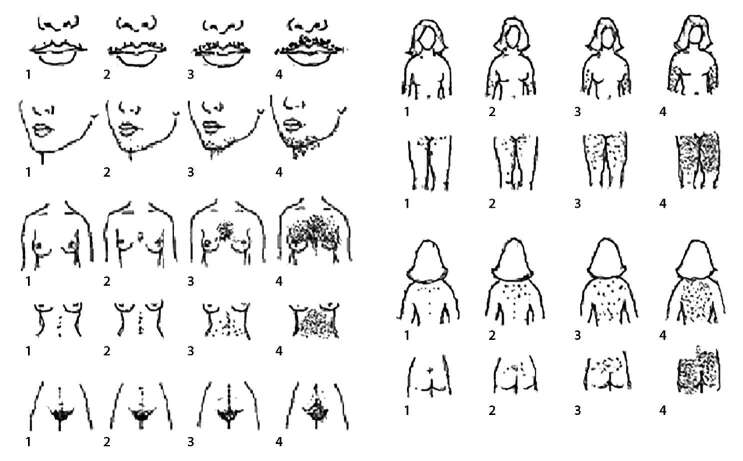
Рисунок 1. Модифицированная шкала Ферримана-Галлвея (Yildizetal., 2010)(цитируется с разрешения авторов).Figure 1. Modified Ferriman-Gallwey scale (Yildizetal., 2010) (quoted with permission of the authors).

## ПРИЛОЖЕНИЕ Г2.

## КРИТЕРИИ ИЗБЫТОЧНОГО ВЕСА И ОЖИРЕНИЯ В РАЗЛИЧНЫХ ПОПУЛЯЦИЯХ

**Table:** Классификация ожирения по ИМТ (ВОЗ, 1997)

Раса	Европеоиды	Азиаты
Избыточный вес	ИМТ=25,0-29,9 кг/м2	ИМТ=23,0-24,9 кг/м2
Ожирение	ИМТ > 30 кг/м2 (39)	ИМТ > 27,5 кг/м2 (39)

Тип массы тела	ИМТ, кг/м2	Риск сопутствующих заболеваний
Дефицит массы тела	<18,5	Низкий (повышен риск других заболеваний)
Нормальная масса тела	18,5-24,9	Обычный
Избыточная масса тела (предожирение)	25,0-29,9	Повышенный
Ожирение I степени	30,0-34,9	Высокий
Ожирение II степени	35,0-39,9	Очень высокий
Ожирение III степени	>40	Чрезвычайно высокий

## ПРИЛОЖЕНИЕ Г3

## ДИФФЕРЕНЦИАЛЬНЫЙ ДИАГНОЗ СПЯ С ДРУГИМИ НОЗОЛОГИЯМИ

**Table:** Заболевания и состояния, некоторые проявления которых совпадают с симптомами СПЯ

Заболевания и состояния	Клинические проявления	Тесты, позволяющие провести дифференциальный диагноз
Беременность	Аменорея (а не олигоменорея), прочие симптомы беременности	Хорионический гонадотропин человека (ХГЧ) в сыворотке крови или в моче (позитивный)
Гипоталамическая аменорея	Аменорея, снижение веса/индекса массы тела (ИМТ), интенсивные физические нагрузки в анамнезе, не характерны клинические признаки гиперандрогении, иногда выявляются мультифолликулярные яичники	Лютеинизирующий гормон (ЛГ) и фолликулостимулирующий гормон (ФСГ) в сыворотке крови (снижены или на нижней границе нормы), Эстрадиол сыворотки крови (снижен)
Преждевременная овариальная недостаточность	Аменорея сочетается с симптомами эстрогенного дефицита, включая приливы жара и урогенитальные симптомы	ФСГ сыворотки крови (повышен), эстрадиол сыворотки крови (снижен)
Андроген- продуцирующие	Вирилизация (включая изменение голоса, андрогенную алопецию, клиторомегалию)	Тестостерон сыворотки крови, ДЭАС сыворотки крови (значительно повышены)
Опухоли	быстрая манифестация симптомов	Ультрасонография яичников Магнитно-резонансная томография (МРТ) надпочечников
Синдром или болезнь Иценко-Кушинга	Наряду с клиническими проявлениями, сходными с СПЯ (ожирение по центральному типу, гиперандрогения, нарушения толерантности к углеводам), имеются более специфические симптомы: часто на лице — «лунообразное» лицо) с одновременным уменьшением верхних и нижних конечностей в обхвате из-за атрофии мышечной и жировой ткани, «матронизм» (яркий румянец цианотического оттенка в совокупности с округлившимися чертами лица), скошенные ягодицы (вследствие атрофии мышц), широкие (часто более 1 см) багрово-фиолетовые стрии на животе, внутренней поверхности бедер и плеч, у женщин — на молочных железах, множественные подкожные кровоизлияния, возникающие даже при незначительных травмах и другие проявления	Свободный кортизол в суточной моче (повышен), кортизол в слюне в ночные часы (повышен), супрессивный ночной тест с дексаметазоном (недостаточная супрессия уровня кортизола в сыворотке крови утром)
Акромегалия	Специфичные симптомы: головная боль, сужение полей зрения, увеличение челюсти, языка, размера обуви и перчаток	Свободный ИФР-1 (инсулиноподобный фактор роста) в сыворотке крови (повышен) МРТ гипофиза

Примеры обоснования диагноза СПЯ

СПЯ (гирсутизм, гиперандрогенемия, олигоановуляция, поликистоз яичников по УЗИ).СПЯ (гирсутизм, олигоановуляция, поликистоз яичников по УЗИ).СПЯ (гиперандрогенемия, олигоановуляция, поликистоз яичников по УЗИ).СПЯ (гирсутизм, поликистоз яичников по УЗИ).СПЯ (гиперандрогенемия, поликистоз яичников по УЗИ).СПЯ (гирсутизм, гиперандрогенемия, поликистоз яичников по УЗИ).СПЯ (олигоановуляция, поликистоз яичников по УЗИ).

## ПРИЛОЖЕНИЕ Б.

АЛГОРИТМЫ ДЕЙСТВИЙ ВРАЧА — см Рубрикатор клинических рекомендаций https://cr.minzdrav.gov.ru/

## References

[cit1] Teede Helena J., Misso Marie L., Costello Michael F., Dokras Anuja, Laven Joop, Moran Lisa, Piltonen Terhi, Norman Robert.J., Andersen Marianne, Azziz Ricardo, Balen Adam, Baye Estifanos, Boyle Jacqueline, Brennan Leah, Broekmans Frank, Dabadghao Preeti, Devoto Luigi, Dewailly Didier, Downes Linda, Fauser Bart, Franks Stephen, Garad Rhonda M., Gibson-Helm Melanie, Harrison Cheryce, Hart Roger, Hawkes Rachel, Hirschberg Angelica, Hoeger Kathleen, Hohmann Femke, Hutchison Samantha, Joham Anju, Johnson Louise, Jordan Cailin, Kulkarni Jayashri, Legro Richard S., Li Rong, Lujan Marla, Malhotra Jaideep, Mansfield Darren, Marsh Kate, McAllister Veryan, Mocanu Edgar, Mol Ben W., Ng Ernest, Oberfield Sharon, Ottey Sasha, Peña Alexia, Qiao Jie, Redman Leanne, Rodgers Raymond, Rombauts Luk, Romualdi Daniela, Shah Duru, Speight Jane, Spritzer Poli Mara, Stener-Victorin Elisabet, Stepto Nigel, Tapanainen Juha S., Tassone Eliza C., Thangaratinam Shakila, Thondan Mala, Tzeng Chii-Ruey, van der Spuy Zephne, Vanky Eszter, Vogiatzi Maria, Wan Angela, Wijeyaratne Chandrika, Witchel Selma, Woolcock Jane, Yildiz Bulent O. (2018). Recommendations from the international evidence-based guideline for the assessment and management of polycystic ovary syndrome. Fertility and Sterility.

[cit2] Goodman Neil F., Cobin Rhoda H., Futterweit Walter, Glueck Jennifer S., Legro Richard S., Carmina Enrico (2015). American Association Of Clinical Endocrinologists, American College Of Endocrinology, And Androgen Excess And Pcos Society Disease State Clinical Review: Guide To The Best Practices In The Evaluation And Treatment Of Polycystic Ovary Syndrome - Part 2. Endocrine Practice.

[cit3] Randeva HS, Tan BK, Weickert MO, et al. Cardiometabolic Aspects of the Polycystic Ovary Syndrome. Endocr Rev. 2012;33(5):812-841. doi: https://doi.org/10.1210/er.2012-1003 22829562 PMC3461136

[cit4] Azziz R, Carmina E, Chen Z, et al. Polycystic ovary syndrome. Nat Rev Dis Prim. 2016;2(1):16057. doi: https://doi.org/10.1038/nrdp.2016.57. 27510637

[cit5] Neven Adriana, Laven Joop, Teede Helena, Boyle Jacqueline (2018). A Summary on Polycystic Ovary Syndrome: Diagnostic Criteria, Prevalence, Clinical Manifestations, and Management According to the Latest International Guidelines. Seminars in Reproductive Medicine.

[cit6] Carmina Enrico, Guastella Ettore, Longo Rosa (2016). Advances in the Diagnosis and Treatment of PCOS. Current Pharmaceutical Design.

[cit7] (2003). Revised 2003 consensus on diagnostic criteria and long-term health risks related to polycystic ovary syndrome (PCOS). Human Reproduction.

[cit8] Spritzer Poli, Barone Carolina, Oliveira Fabiana (2016). Hirsutism in Polycystic Ovary Syndrome: Pathophysiology and Management. Current Pharmaceutical Design.

[cit9] Ezeh Uche, Huang Andy, Landay Melanie, Azziz Ricardo (2018). Long-Term Response of Hirsutism and Other Hyperandrogenic Symptoms to Combination Therapy in Polycystic Ovary Syndrome. Journal of Women's Health.

[cit10] Lizneva Daria, Gavrilova-Jordan Larisa, Walker Walidah, Azziz Ricardo (2016). Androgen excess: Investigations and management. Best Practice & Research Clinical Obstetrics & Gynaecology.

[cit11] Escobar-Morreale H.F., Carmina E., Dewailly D., Gambineri A., Kelestimur F., Moghetti P., Pugeat M., Qiao J., Wijeyaratne C.N., Witchel S.F., Norman R.J. (2011). Epidemiology, diagnosis and management of hirsutism: a consensus statement by the Androgen Excess and Polycystic Ovary Syndrome Society. Human Reproduction Update.

[cit12] Legro Richard S., Schlaff William D., Diamond Michael P., Coutifaris Christos, Casson Peter R., Brzyski Robert G., Christman Gregory M., Trussell J. C., Krawetz Stephen A., Snyder Peter J., Ohl Dana, Carson Sandra A., Steinkampf Michael P., Carr Bruce R., McGovern Peter G., Cataldo Nicholas A., Gosman Gabriella G., Nestler John E., Myers Evan R., Santoro Nanette, Eisenberg Esther, Zhang Meizhuo, Zhang Heping (2010). Total Testosterone Assays in Women with Polycystic Ovary Syndrome: Precision and Correlation with Hirsutism. The Journal of Clinical Endocrinology & Metabolism.

[cit13] Lause Michael, Kamboj Alisha, Fernandez Faith Esteban (2017). Dermatologic manifestations of endocrine disorders. Translational Pediatrics.

[cit14] Melibary Yaser Taha (2018). Hidradenitis suppurativa in a patient with hyperandrogenism, insulin-resistance and acanthosis nigricans (HAIR-AN syndrome). Dermatology Reports.

[cit15] Keen MohammadAbid, Shah IffatHassan, Sheikh Gousia (2017). Cutaneous manifestations of polycystic ovary syndrome: A cross-sectional clinical study. Indian Dermatology Online Journal.

[cit16] Rodgers Raymond J, Avery Jodie C, Moore Vivienne M, Davies Michael J, Azziz Ricardo, Stener-Victorin Elisabet, Moran Lisa J, Robertson Sarah A, Stepto Nigel K, Norman Robert J, Teede Helena J (2019). Complex diseases and co-morbidities: polycystic ovary syndrome and type 2 diabetes mellitus. Endocrine Connections.

[cit17] Legro Richard (2012). Obesity and PCOS: Implications for Diagnosis and Treatment. Seminars in Reproductive Medicine.

[cit18] Glueck Charles J., Goldenberg Naila (2018). Characteristics of obesity in polycystic ovary syndrome: Etiology, treatment, and genetics. Metabolism.

[cit19] Pasquali Renato, Oriolo Claudia (2019). Obesity and Androgens in Women. Frontiers of Hormone Research.

[cit20] Zheng Sai-Hua, Li Xue-Lian (2015). Visceral adiposity index as a predictor of clinical severity and therapeutic outcome of PCOS. Gynecological Endocrinology.

[cit21] Gibson-Helm Melanie, Teede Helena, Dunaif Andrea, Dokras Anuja (2016). Delayed diagnosis and a lack of information associated with dissatisfaction in women with polycystic ovary syndrome. The Journal of Clinical Endocrinology & Metabolism.

[cit22] Dokras Anuja, Saini Shailly, Gibson-Helm Melanie, Schulkin Jay, Cooney Laura, Teede Helena (2017). Gaps in knowledge among physicians regarding diagnostic criteria and management of polycystic ovary syndrome. Fertility and Sterility.

[cit23] Balen Adam H., Morley Lara C., Misso Marie, Franks Stephen, Legro Richard S., Wijeyaratne Chandrika N., Stener-Victorin Elisabet, Fauser Bart C.J.M., Norman Robert J., Teede Helena (2016). The management of anovulatory infertility in women with polycystic ovary syndrome: an analysis of the evidence to support the development of global WHO guidance. Human Reproduction Update.

[cit24] Wild Robert A., Carmina Enrico, Diamanti-Kandarakis Evanthia, Dokras Anuja, Escobar-Morreale Hector F., Futterweit Walter, Lobo Rogerio, Norman Robert J., Talbott Evelyn, Dumesic Daniel A. (2010). Assessment of Cardiovascular Risk and Prevention of Cardiovascular Disease in Women with the Polycystic Ovary Syndrome: A Consensus Statement by the Androgen Excess and Polycystic Ovary Syndrome (AE-PCOS) Society. The Journal of Clinical Endocrinology & Metabolism.

[cit25] Andersen Marianne, Glintborg Dorte (2018). Diagnosis and follow-up of type 2 diabetes in women with PCOS: a role for OGTT?. European Journal of Endocrinology.

[cit26] Anagnostis Panagiotis, Tarlatzis Basil C., Kauffman Robert P. (2017). Polycystic ovarian syndrome (PCOS): Long-term metabolic consequences. Metabolism.

[cit27] Nolan Christopher J, Prentki Marc (2019). Insulin resistance and insulin hypersecretion in the metabolic syndrome and type 2 diabetes: Time for a conceptual framework shift. Diabetes and Vascular Disease Research.

[cit28] Pelanis Rasa, Mellembakken Jan Roar, Sundström-Poromaa Inger, Ravn Pernille, Morin-Papunen Laure, Tapanainen Juha S, Piltonen Terhi, Puurunen Johanna, Hirschberg Angelica Lindén, Fedorcsak Peter, Andersen Marianne, Glintborg Dorte (2017). The prevalence of Type 2 diabetes is not increased in normal-weight women with PCOS. Human Reproduction.

[cit29] Condorelli Rosita A., Calogero Aldo E., Di Mauro Maurizio, La Vignera Sandro (2017). PCOS and diabetes mellitus: from insulin resistance to altered beta pancreatic function, a link in evolution. Gynecological Endocrinology.

[cit30] Jeanes Yvonne M., Reeves Sue (2017). Metabolic consequences of obesity and insulin resistance in polycystic ovary syndrome: diagnostic and methodological challenges. Nutrition Research Reviews.

[cit31] Mohammad MB, Seghinsara AM. Polycystic ovary syndrome (PCOS), diagnostic criteria, and AMH. Asian Pacific J Cancer Prev. 2017;18(1):17-21. doi: https://doi.org/10.22034/APJCP.2017.18.1.17 PMC5563096 28240001

[cit32] Teede Helena, Misso Marie, Tassone Eliza C., Dewailly Didier, Ng Ernest Hy, Azziz Ricardo, Norman Robert J., Andersen Marianne, Franks Stephen, Hoeger Kathleen, Hutchison Samantha, Oberfield Sharon, Shah Duru, Hohmann Femke, Ottey Sasha, Dabadghao Preeti, Laven Joop S.E. (2019). Anti-Müllerian Hormone in PCOS: A Review Informing International Guidelines. Trends in Endocrinology & Metabolism.

[cit33] Garg Deepika, Tal Reshef (2016). The role of AMH in the pathophysiology of polycystic ovarian syndrome. Reproductive BioMedicine Online.

[cit34] Sova Henri, Unkila-Kallio Leila, Tiitinen Aila, Hippeläinen Maritta, Perheentupa Antti, Tinkanen Helena, Puukka Katri, Bloigu Risto, Piltonen Terhi, Tapanainen Juha S., Morin-Papunen Laure (2019). Hormone profiling, including anti-Müllerian hormone (AMH), for the diagnosis of polycystic ovary syndrome (PCOS) and characterization of PCOS phenotypes. Gynecological Endocrinology.

[cit35] Yildiz B. O., Bozdag G., Yapici Z., Esinler I., Yarali H. (2012). Prevalence, phenotype and cardiometabolic risk of polycystic ovary syndrome under different diagnostic criteria. Human Reproduction.

[cit36] Alberti K.G.M.M., Eckel Robert H., Grundy Scott M., Zimmet Paul Z., Cleeman James I., Donato Karen A., Fruchart Jean-Charles, James W. Philip T., Loria Catherine M., Smith Sidney C. (2009). Harmonizing the Metabolic Syndrome. Circulation.

[cit37] Kakoly N S, Khomami M B, Joham A E, Cooray S D, Misso M L, Norman R J, Harrison C L, Ranasinha S, Teede H J, Moran L J (2018). Ethnicity, obesity and the prevalence of impaired glucose tolerance and type 2 diabetes in PCOS: a systematic review and meta-regression. Human Reproduction Update.

[cit38] Legro Richard S., Arslanian Silva A., Ehrmann David A., Hoeger Kathleen M., Murad M. Hassan, Pasquali Renato, Welt Corrine K. (2013). Diagnosis and Treatment of Polycystic Ovary Syndrome: An Endocrine Society Clinical Practice Guideline. The Journal of Clinical Endocrinology & Metabolism.

[cit39] Rosenfield Robert L. (2014). The Polycystic Ovary Morphology-Polycystic Ovary Syndrome Spectrum. Journal of Pediatric and Adolescent Gynecology.

[cit40] Dewailly D., Lujan M. E., Carmina E., Cedars M. I., Laven J., Norman R. J., Escobar-Morreale H. F. (2013). Definition and significance of polycystic ovarian morphology: a task force report from the Androgen Excess and Polycystic Ovary Syndrome Society. Human Reproduction Update.

[cit41] Zhu Ruo-Yan, Wong Yee-Chee, Yong Eu-Leong (2016). Sonographic evaluation of polycystic ovaries. Best Practice & Research Clinical Obstetrics & Gynaecology.

[cit42] Federal'nye klinicheskie rekomendatsii. Bolezn' Itsenko-Kushinga. 2016.

[cit43] Federal'nye klinicheskie rekomendatsii. Vrozhdennaya disfunktsiya kory nadpochechnikov u vzroslykh. 2016.

[cit44] Federal'nye klinicheskie rekomendatsii. Giperprolaktinemiya. 2016.

[cit45] Alexander Erik K., Pearce Elizabeth N., Brent Gregory A., Brown Rosalind S., Chen Herbert, Dosiou Chrysoula, Grobman William A., Laurberg Peter, Lazarus John H., Mandel Susan J., Peeters Robin P., Sullivan Scott (2017). 2017 Guidelines of the American Thyroid Association for the Diagnosis and Management of Thyroid Disease During Pregnancy and the Postpartum. Thyroid.

[cit46] Azziz R, Carmina E, Dewailly D, et al. The Androgen Excess and PCOS Society criteria for the polycystic ovary syndrome: the complete task force report. Fertil Steril. 2009;91(2):456-488. doi: https://doi.org/10.1016/j.fertnstert.2008.06.035 18950759

[cit47] Osibogun Olatokunbo, Ogunmoroti Oluseye, Michos Erin D. (2019). Polycystic ovary syndrome and cardiometabolic risk: Opportunities for cardiovascular disease prevention. Trends in Cardiovascular Medicine.

[cit48] Wild Robert A. (2011). Dyslipidemia in PCOS. Steroids.

[cit49] Papadakis Georgios, Kandaraki Eleni, Papalou Olga, Vryonidou Andromachi, Diamanti‑Kandarakis Evanthia (2021). Is cardiovascular risk in women with PCOS a real risk? Current insights. Minerva Endocrinology.

[cit50] Meun Cindy, Gunning Marlise N, Louwers Yvonne V, Peters Henrike, Roos‐Hesselink Jolien, Roeters van Lennep Jeanine, Rueda Ochoa Oscar‐Leonel, Appelman Yolande, Lambalk Nils, Boersma Eric, Kavousi Maryam, Fauser Bart CJM, Laven Joop SE, Baart Sara, Benschop Laura, Brouwers Laura, Budde Ricardo, Cannegieter Suzanne, Dam Veerle, Eijkemans Rene, Ferrari Michel, Franx Arie, de Groot Christianne, Hoek Annemieke, Koffijberg Erik, Koster Wendy, Kruit Mark, Lagerweij Giske, Linstra Katie, van der Lugt Aad, Maas Angela, Maassen van den Brink Antoinette, Middeldorp Saskia, Moons Karel GM, van Rijn Bas, Scheres Luuk, van der Schouw Yvonne T., Steegers Eric, Steegers Regine, Terwindt Gisela, Velthuis Birgitta, Wermer Marieke, Zick Bart, Zoet Gerbrand (2019). The cardiovascular risk profile of middle‐aged women with polycystic ovary syndrome. Clinical Endocrinology.

[cit51] Delitala AP, Capobianco G, Delitala G, et al. Polycystic ovary syndrome, adipose tissue and metabolic syndrome. Arch Gynecol Obstet. 2017;296(3):405-419. doi: https://doi.org/10.1007/s00404-017-4429-2 28643028

[cit52] Damone Anna L., Joham Anju E., Loxton Deborah, Earnest Arul, Teede Helena J., Moran Lisa J. (2018). Depression, anxiety and perceived stress in women with and without PCOS: a community-based study. Psychological Medicine.

[cit53] Cooney Laura G., Dokras Anuja (2017). Depression and Anxiety in Polycystic Ovary Syndrome: Etiology and Treatment. Current Psychiatry Reports.

[cit54] Rodriguez-Paris Daniel, Remlinger-Molenda Agnieszka, Kurzawa Rafał, Głowińska Aleksandra, Spaczyński Robert, Rybakowski Filip, Pawełczyk Leszek, Banaszewska Beata (2019). The occurrence of psychiatric disorders in women with polycystic ovary syndrome. Psychiatria Polska.

[cit55] Brutocao Claire, Zaiem Feras, Alsawas Mouaz, Morrow Allison S., Murad M. Hassan, Javed Asma (2018). Psychiatric disorders in women with polycystic ovary syndrome: a systematic review and meta-analysis. Endocrine.

[cit56] Lim Siew S, Hutchison Samantha K, Van Ryswyk Emer, Norman Robert J, Teede Helena J, Moran Lisa J (2019). Lifestyle changes in women with polycystic ovary syndrome. Cochrane Database of Systematic Reviews.

[cit57] Oberg Emma, Gidlöf Sebastian, Jakson Ivika, Mitsell Marja, Tollet Egnell Petra, Hirschberg Angelica Lindén (2018). Improved menstrual function in obese women with polycystic ovary syndrome after behavioural modification intervention-A randomized controlled trial. Clinical Endocrinology.

[cit58] dos Santos Isis Kelly, Ashe Maureen C., Cobucci Ricardo Ney, Soares Gustavo Mafaldo, de Oliveira Maranhão Tecia Maria, Dantas Paulo Moreira Silva (2020). The effect of exercise as an intervention for women with polycystic ovary syndrome. Medicine.

[cit59] Teede Helena, Tassone Eliza C., Piltonen Terhi, Malhotra Jaideep, Mol Ben W., Peña Alexia, Witchel Selma F., Joham Anju, McAllister Veryan, Romualdi Daniela, Thondan Mala, Costello Michael, Misso Marie L. (2019). Effect of the combined oral contraceptive pill and/or metformin in the management of polycystic ovary syndrome: A systematic review with meta‐analyses. Clinical Endocrinology.

[cit60] Wang Qiu-Yi, Song Yong, Huang Wei, Xiao Li, Wang Qiu-Shi, Feng Gui-Mei (2016). Comparison of Drospirenone- with Cyproterone Acetate-Containing Oral Contraceptives, Combined with Metformin and Lifestyle Modifications in Women with Polycystic Ovary Syndrome and Metabolic Disorders. Chinese Medical Journal.

[cit61] Feng Wei, Jia Yan-Yan, Zhang Dong-Ya, Shi Hui-Rong (2015). Management of polycystic ovarian syndrome with Diane-35 or Diane-35 plus metformin. Gynecological Endocrinology.

[cit62] Shah Aesha, Dodson William C, Kris-Etherton Penny M, Kunselman Allen R, Stetter Christy M, Gnatuk Carol L, Estes Stephanie J, Allison Kelly C, Sarwer David B, Sluss Patrick M, Coutifaris Christos, Dokras Anuja, Legro Richard S (2020). Effects of Oral Contraception and Lifestyle Modification on Incretins and TGF-ß Superfamily Hormones in PCOS. The Journal of Clinical Endocrinology & Metabolism.

[cit63] Amiri Mina, Nahidi Fatemeh, Yarandi Razieh Bidhendi, Khalili Davood, Tohidi Maryam, Tehrani Fahimeh Ramezani (2020). Effects of oral contraceptives on the quality of life of women with polycystic ovary syndrome: a crossover randomized controlled trial. Health and Quality of Life Outcomes.

[cit64] Fonseka S, Wijeyaratne CN, Gawarammana IB, et al. Effectiveness of Low-dose Ethinylestradiol/Cyproterone Acetate and Ethinylestradiol/Desogestrel with and without Metformin on Hirsutism in Polycystic Ovary Syndrome: A Randomized, Double-blind, Triple-dummy Study. J Clin Aesthet Dermatol. 2020;13(7):18-23. PMC7492016 32983332

[cit65] World Health Organization. [Internet]. 2015 Quick Reference Chart for the WHO Medical Eligibility Criteria for Contraceptive Use. Adapted from Medical Eligibility Criteria for Contraceptive Use, 5th Edition 2015. Available from: https://www.fhi360.org/sites/default/files/media/documents/chart-medical-eligibility-contraceptives-english.pdf.

[cit66] Tay Chau T., Joham Anju E., Hiam Danielle S., Gadalla Moustafa A., Pundir Jyotsna, Thangaratinam Shakila, Teede Helena J., Moran Lisa J. (2018). Pharmacological and surgical treatment of nonreproductive outcomes in polycystic ovary syndrome: An overview of systematic reviews. Clinical Endocrinology.

[cit67] Bayram N. (2004). Using an electrocautery strategy or recombinant follicle stimulating hormone to induce ovulation in polycystic ovary syndrome: randomised controlled trial. BMJ.

[cit68] Fraison Eloise, Kostova Elena, Moran Lisa J, Bilal Sophia, Ee Carolyn C, Venetis Christos, Costello Michael F (2020). Metformin versus the combined oral contraceptive pill for hirsutism, acne, and menstrual pattern in polycystic ovary syndrome. Cochrane Database of Systematic Reviews.

[cit69] Jensterle Mojca, Kravos Nika Aleksandra, Ferjan Simona, Goricar Katja, Dolzan Vita, Janez Andrej (2020). Long-term efficacy of metformin in overweight-obese PCOS: longitudinal follow-up of retrospective cohort. Endocrine Connections.

[cit70] Panda Soumya Ranjan, Jain Madhu, Jain Shuchi, Saxena Riden, Hota Smrutismita (2018). Effect of Orlistat Versus Metformin in Various Aspects of Polycystic Ovarian Syndrome: A Systematic Review of Randomized Control Trials. The Journal of Obstetrics and Gynecology of India.

[cit71] Abdalla Mohammed Altigani, Deshmukh Harshal, Atkin Stephen, Sathyapalan Thozhukat (2020). A review of therapeutic options for managing the metabolic aspects of polycystic ovary syndrome. Therapeutic Advances in Endocrinology and Metabolism.

[cit72] Showell Marian G, Mackenzie-Proctor Rebecca, Jordan Vanessa, Hodgson Ruth, Farquhar Cindy (2018). Inositol for subfertile women with polycystic ovary syndrome. Cochrane Database of Systematic Reviews.

[cit73] Wang Rui, Li Wentao, Bordewijk Esmée M, Legro Richard S, Zhang Heping, Wu Xiaoke, Gao Jingshu, Morin-Papunen Laure, Homburg Roy, König Tamar E, Moll Etelka, Kar Sujata, Huang Wei, Johnson Neil P, Amer Saad A, Vegetti Walter, Palomba Stefano, Falbo Angela, Özmen Ülkü, Nazik Hakan, Williams Christopher D, Federica Grasso, Lord Jonathan, Sahin Yilmaz, Bhattacharya Siladitya, Norman Robert J, van Wely Madelon, Mol Ben Willem (2019). First-line ovulation induction for polycystic ovary syndrome: an individual participant data meta-analysis. Human Reproduction Update.

[cit74] Morley Lara C, Tang Thomas, Yasmin Ephia, Norman Robert J, Balen Adam H (2017). Insulin-sensitising drugs (metformin, rosiglitazone, pioglitazone, D-chiro-inositol) for women with polycystic ovary syndrome, oligo amenorrhoea and subfertility. Cochrane Database of Systematic Reviews.

[cit75] Sharpe Abigail, Morley Lara C, Tang Thomas, Norman Robert J, Balen Adam H (2019). Metformin for ovulation induction (excluding gonadotrophins) in women with polycystic ovary syndrome. Cochrane Database of Systematic Reviews.

[cit76] Wang Rui, Kim Bobae V, van Wely Madelon, Johnson Neil P, Costello Michael F, Zhang Hanwang, Ng Ernest Hung Yu, Legro Richard S, Bhattacharya Siladitya, Norman Robert J, Mol Ben Willem J (2017). Treatment strategies for women with WHO group II anovulation: systematic review and network meta-analysis. BMJ.

[cit77] Weiss Nienke S, Kostova Elena, Nahuis Marleen, Mol Ben Willem J, van der Veen Fulco, van Wely Madelon (2019). Gonadotrophins for ovulation induction in women with polycystic ovary syndrome. Cochrane Database of Systematic Reviews.

[cit78] Bordewijk Esmée M, Ng Ka Ying Bonnie, Rakic Lidija, Mol Ben Willem J, Brown Julie, Crawford Tineke J, van Wely Madelon (2020). Laparoscopic ovarian drilling for ovulation induction in women with anovulatory polycystic ovary syndrome. Cochrane Database of Systematic Reviews.

[cit79] Lepine Sam, Jo Junyoung, Metwally Mostafa, Cheong Ying C (2017). Ovarian surgery for symptom relief in women with polycystic ovary syndrome. Cochrane Database of Systematic Reviews.

[cit80] Christ Jacob P., Falcone Tommaso (2018). Bariatric Surgery Improves Hyperandrogenism, Menstrual Irregularities, and Metabolic Dysfunction Among Women with Polycystic Ovary Syndrome (PCOS). Obesity Surgery.

[cit81] Singh Devender, Arumalla Kirit, Aggarwal Sandeep, Singla Vitish, Ganie Ashraf, Malhotra Neena (2020). Impact of Bariatric Surgery on Clinical, Biochemical, and Hormonal Parameters in Women with Polycystic Ovary Syndrome (PCOS). Obesity Surgery.

[cit82] (2008). Consensus on infertility treatment related to polycystic ovary syndrome. Human Reproduction.

[cit83] Yaylalı Aslı, Bakacak Murat, Bakacak Zeyneb (2020). The efficacy of different insulin-sensitizing agents on treatment outcomes in patients with polycystic ovary syndrome who underwent in-vitro fertilization: A retrospective, record-based, comparative study. Journal of Gynecology Obstetrics and Human Reproduction.

[cit84] Bordewijk Esmée M, Nahuis Marleen, Costello Michael F, Van der Veen Fulco, Tso Leopoldo O, Mol Ben Willem J, van Wely Madelon (2017). Metformin during ovulation induction with gonadotrophins followed by timed intercourse or intrauterine insemination for subfertility associated with polycystic ovary syndrome. Cochrane Database of Systematic Reviews.

[cit85] Tso Leopoldo O, Costello Michael F, Albuquerque Luiz Eduardo T, Andriolo Regis B, Macedo Cristiane R (2020). Metformin treatment before and during IVF or ICSI in women with polycystic ovary syndrome. Cochrane Database of Systematic Reviews.

[cit86] Wu Yiqing, Tu Mixue, Huang Yun, Liu Yifeng, Zhang Dan (2020). Association of Metformin With Pregnancy Outcomes in Women With Polycystic Ovarian Syndrome Undergoing In Vitro Fertilization. JAMA Network Open.

[cit87] Bahri Khomami Mahnaz, Joham Anju E., Boyle Jacqueline A., Piltonen Terhi, Arora Chavy, Silagy Michael, Misso Marie L., Teede Helena J., Moran Lisa J. (2019). The role of maternal obesity in infant outcomes in polycystic ovary syndrome—A systematic review, meta‐analysis, and meta‐regression. Obesity Reviews.

[cit88] Bahri Khomami Mahnaz, Joham Anju E., Boyle Jacqueline A., Piltonen Terhi, Silagy Michael, Arora Chavy, Misso Marie L., Teede Helena J., Moran Lisa J. (2019). Increased maternal pregnancy complications in polycystic ovary syndrome appear to be independent of obesity—A systematic review, meta‐analysis, and meta‐regression. Obesity Reviews.

[cit89] Gunning Marlise N, Sir Petermann Teresa, Crisosto Nicolas, van Rijn Bas B, de Wilde Marlieke A, Christ Jacob P, Uiterwaal C S P M, de Jager Wilco, Eijkemans Marinus J C, Kunselman Allen R, Legro Richard S, Fauser Bart C J M (2019). Cardiometabolic health in offspring of women with PCOS compared to healthy controls: a systematic review and individual participant data meta-analysis. Human Reproduction Update.

[cit90] Rodgers Raymond J, Avery Jodie C, Moore Vivienne M, Davies Michael J, Azziz Ricardo, Stener-Victorin Elisabet, Moran Lisa J, Robertson Sarah A, Stepto Nigel K, Norman Robert J, Teede Helena J (2019). Complex diseases and co-morbidities: polycystic ovary syndrome and type 2 diabetes mellitus. Endocrine Connections.

[cit91] Horowitz Eran, Weissman Ariel (2020). The stair-step approach in treatment of anovulatory PCOS patients. Therapeutic Advances in Reproductive Health.

[cit92] Gadalla MA, Norman RJ, Tay CT, et al. Medical and surgical treatment of reproductive outcomes in polycystic ovary syndrome: An overview of systematic reviews. Int J Fertil Steril. 2020;13(4):257-270. doi: https://doi.org/10.22074/ijfs.2020.5608 31710185 PMC6875858

[cit93] Obeid Rima, Schön Christiane, Wilhelm Manfred, Pietrzik Klaus, Pilz Stefan (2018). Dietary and lifestyle predictors of folate insufficiency in non-supplemented German women. International Journal of Food Sciences and Nutrition.

[cit94] Daly Leslie E. (2011). Folate Levels and Neural Tube Defects. JAMA.

[cit95] Moreno Luis A., Gottrand Frédéric, Huybrechts Inge, Ruiz Jonatan R., González-Gross Marcela, DeHenauw Stefaan (2014). Nutrition and Lifestyle in European Adolescents: The HELENA (Healthy Lifestyle in Europe by Nutrition in Adolescence) Study. Advances in Nutrition.

[cit96] Osterhues Anja, Holzgreve Wolfgang, Michels Karin B (2009). Shall we put the world on folate?. The Lancet.

[cit97] Escobar-Morreale HF, Luque-Ramírez M, González F. Circulating inflammatory markers in polycystic ovary syndrome: a systematic review and metaanalysis. Fertil Steril. 2011;95(3):1048-1058.e2. doi: https://doi.org/10.1016/j.fertnstert.2010.11.036 PMC3079565 21168133

[cit98] Carvalho Maria João, Subtil Simone, Rodrigues Ângela, Oliveira Joana, Figueiredo-Dias Margarida (2019). Controversial association between polycystic ovary syndrome and breast cancer. European Journal of Obstetrics & Gynecology and Reproductive Biology.

[cit99] Meczekalski Blazej, Pérez-Roncero Gonzalo R., López-Baena María T., Chedraui Peter, Pérez-López Faustino R. (2020). The polycystic ovary syndrome and gynecological cancer risk. Gynecological Endocrinology.

[cit100] Wen Yaokai, Wu Xiangrong, Peng Haoxin, Li Caichen, Jiang Yu, Su Zixuan, Liang Hengrui, Liu Jun, He Jianxing, Liang Wenhua (2020). Breast cancer risk in patients with polycystic ovary syndrome: a Mendelian randomization analysis. Breast Cancer Research and Treatment.

[cit101] Belenkaya L.V. (2018). Criteria of obesity for Asian population. Literature review. Acta Biomedica Scientifica.

[cit102] Peña Alexia S., Witchel Selma F., Hoeger Kathleen M., Oberfield Sharon E., Vogiatzi Maria G., Misso Marie, Garad Rhonda, Dabadghao Preeti, Teede Helena (2020). Adolescent polycystic ovary syndrome according to the international evidence-based guideline. BMC Medicine.

[cit103] Aversa Antonio, La Vignera Sandro, Rago Rocco, Gambineri Alessandra, Nappi Rossella E., Calogero Aldo E., Ferlin Alberto (2020). Fundamental Concepts and Novel Aspects of Polycystic Ovarian Syndrome: Expert Consensus Resolutions. Frontiers in Endocrinology.

[cit104] Kazerooni Talieh, Asadi Nasrin, Dehbashi Sedigheh, Zolghadri Jaleh (2008). Effect of folic acid in women with and without insulin resistance who have hyperhomocysteinemic polycystic ovary syndrome. International Journal of Gynecology & Obstetrics.

[cit105] Mondal Kalyani, Chakraborty Pratip, Kabir Syed N. (2018). Hyperhomocysteinemia and hyperandrogenemia share PCSK9-LDLR pathway to disrupt lipid homeostasis in PCOS. Biochemical and Biophysical Research Communications.

[cit106] Escobar-Morreale HF. Polycystic ovary syndrome: definition, aetiology, diagnosis and treatment. Nat Rev Endocrinol. 2018;14(5):270-284. doi: https://doi.org/10.1038/nrendo.2018.24 29569621

[cit107] Glintborg Dorte, Rubin Katrine Hass, Nybo Mads, Abrahamsen Bo, Andersen Marianne (2018). Cardiovascular disease in a nationwide population of Danish women with polycystic ovary syndrome. Cardiovascular Diabetology.

[cit108] Farquhar Cindy, Brown Julie, Marjoribanks Jane (2012). Laparoscopic drilling by diathermy or laser for ovulation induction in anovulatory polycystic ovary syndrome. Cochrane Database of Systematic Reviews.

[cit109] Lim S. S., Norman R. J., Davies M. J., Moran L. J. (2012). The effect of obesity on polycystic ovary syndrome: a systematic review and meta-analysis. Obesity Reviews.

[cit110] Wild Sarah, Pierpoint Tracey, Jacobs Howard, McKeigue Paul (2007). Long-term consequences of polycystic ovary syndrome: Results of a 31 year follow-up study. Human Fertility.

[cit111] van Zuuren EJ, Fedorowicz Z, Carter B, Pandis N. Interventions for hirsutism (excluding laser and photoepilation therapy alone). Cochrane Database Syst Rev. 2015;4. doi: https://doi.org/10.1002/14651858.CD010334.pub2 PMC6481758 25918921

[cit112] Abu Hashim Hatem, Foda Osama, El Rakhawy Mohamed (2018). Unilateral or bilateral laparoscopic ovarian drilling in polycystic ovary syndrome: a meta-analysis of randomized trials. Archives of Gynecology and Obstetrics.

[cit113] Mulder CL, Lassi ZS, Grieger JA, Ali A, Jankovic‐Karasoulos T, Roberts CT, Andraweera PH (2020). Cardio‐metabolic risk factors among young infertile women: a systematic review and meta‐analysis. BJOG: An International Journal of Obstetrics & Gynaecology.

[cit114] Kahal Hassan, Kyrou Ioannis, Uthman Olalekan A., Brown Anna, Johnson Samantha, Wall Peter D. H., Metcalfe Andrew, Parr David G., Tahrani Abd A., Randeva Harpal S. (2019). The prevalence of obstructive sleep apnoea in women with polycystic ovary syndrome: a systematic review and meta-analysis. Sleep and Breathing.

[cit115] Oriolo C., Fanelli F., Castelli S., Mezzullo M., Altieri P., Corzani F., Pelusi C., Repaci A., Di Dalmazi G., Vicennati V., Baldazzi L., Menabò S., Dormi A., Nardi E., Brillanti G., Pasquali R., Pagotto U., Gambineri A. (2020). Steroid biomarkers for identifying non-classic adrenal hyperplasia due to 21-hydroxylase deficiency in a population of PCOS with suspicious levels of 17OH-progesterone. Journal of Endocrinological Investigation.

[cit116] Jonard Sophie, Robert Yann, Dewailly Didier (2005). Revisiting the ovarian volume as a diagnostic criterion for polycystic ovaries. Human Reproduction.

[cit117] Pinola P., Morin-Papunen L. C., Bloigu A., Puukka K., Ruokonen A., Jarvelin M.- R., Franks S., Tapanainen J. S., Lashen H. (2014). Anti-Mullerian hormone: correlation with testosterone and oligo- or amenorrhoea in female adolescence in a population-based cohort study. Human Reproduction.

[cit118] Moran Lisa J., Misso Marie L., Wild Robert A., Norman Robert J. (2010). Impaired glucose tolerance, type 2 diabetes and metabolic syndrome in polycystic ovary syndrome: a systematic review and meta-analysis. Human Reproduction Update.

[cit119] Tosi Flavia, Fiers Tom, Kaufman Jean-Marc, Dall'Alda Marlene, Moretta Rossella, Giagulli Vito Angelo, Bonora Enzo, Moghetti Paolo (2015). Implications of Androgen Assay Accuracy in the Phenotyping of Women With Polycystic Ovary Syndrome. The Journal of Clinical Endocrinology & Metabolism.

[cit120] Steck T., Wernze H. (2009). Ist die Bestimmung des «freien Androgenindexes» zum Hormonscreening bei polyzystischen Ovarien sinnvoll?. Gynäkologisch-geburtshilfliche Rundschau.

[cit121] Kollmann M., Martins W. P., Lima M. L. S., Craciunas L., Nastri C. O., Richardson A., Raine-Fenning N. (2016). Strategies for improving outcome of assisted reproduction in women with polycystic ovary syndrome: systematic review and meta-analysis. Ultrasound in Obstetrics & Gynecology.

[cit122] Zaenglein AL, Pathy AL, Schlosser BJ, et al. Guidelines of care for the management of acne vulgaris. J Am Acad Dermatol. 2016;74(5):945-973.e33. doi: https://doi.org/10.1016/j.jaad.2015.12.037 26897386

[cit123] Barrionuevo Patricia, Nabhan Mohammed, Altayar Osama, Wang Zhen, Erwin Patricia J, Asi Noor, Martin Kathryn A, Murad M Hassan (2018). Treatment Options for Hirsutism: A Systematic Review and Network Meta-Analysis. The Journal of Clinical Endocrinology & Metabolism.

[cit124] Derewianka-Polak Magdalena, Polak Grzegorz, Tkaczuk-Włach Joanna, Gerhant Aneta, Olajossy Marcin (2020). Polycystic ovary syndrome and mental disorders – discussion on the recommendations of the European Society of Human Reproduction and Embryology (ESHRE). Current Problems of Psychiatry.

[cit125] Zeinalzadeh M., Basirat Z., Esmailpour M. Efficacy of letrozole in ovulation induction compared to that of clomiphene citrate in patients with polycystic ovarian syndrome. The Journal of reproductive medicine. 2010;55(1-2):36-40. 20337206

